# Disruption as Antecedent to New Business Startup: A Study of Entrepreneurship Using the Understanding America Study

**DOI:** 10.1093/geroni/igaa057.1488

**Published:** 2020-12-16

**Authors:** Felichism Kabo, Stewart Thornhill, Elizabeth Isele

**Affiliations:** 1 University of Michigan, Ann Arbor, Michigan, United States; 2 GIEE (Global Institute for Experienced Entrepreneurship), North Yarmouth, Maine, United States

## Abstract

We use data from a nationally representative study to examine antecedents of entrepreneurial activity in the US among younger (< age 50) and older (≥ age 50) adults using Shapero’s and Sokol’s Model of the “Entrepreneurial Event” (hereafter SEE). The “entrepreneurial event” here refers to the initiation of entrepreneurial behavior. The SEE approach assumes that individuals default to inertia until their lives are disrupted by exogenous factors such as life-changing events (Shapero & Sokol, 1982). The disruptor could be either positive or negative, and has the net effect of driving the individual to reconsider entrepreneurship as a viable opportunity (Krueger & Brazeal, 1994; Krueger & Day, 2010). We examine the correlation between negative disruptions and a person initiating entrepreneurial behavior (starting a new business), including whether the process is similar across younger and older adults. Using data collected in 2014 and 2019 from 3,586 individuals in the Understanding America Study panel, we run survey Cox proportional hazards models to analyze the effects of negative disruptions (getting separated or divorced) on starting a new business over a five-year period starting in 2014. We found that negative disruptions have a significant, positive effect but only among older adults. Further, the magnitude of that effect is about 3-7 times that of younger adults. Our findings support the validity of the SEE approach in advancing our understanding of the transition of individuals from potential to actual entrepreneurs. However, the findings suggest the SEE approach better explains this process in older rather than younger adults.

